# Insights into plant regeneration: cellular pathways and DNA methylation dynamics

**DOI:** 10.1007/s00299-024-03216-9

**Published:** 2024-04-18

**Authors:** Seunga Lee, Young Seo Park, Ji Hoon Rhee, Hyojeong Chu, Jennifer M. Frost, Yeonhee Choi

**Affiliations:** 1 Department of Biological Sciences, Seoul National University, Seoul, Korea; 2 Research Center for Plant Plasticity, Seoul National University, Seoul, Korea; 3 The Research Institute of Basic Sciences, Seoul National University, Seoul, Korea; 4 Genomics and Child Health, The Blizard Institute, Queen Mary University of London, London, UK

**Keywords:** Pluripotency, Dedifferentiation, In vitro regeneration, De novo organogenesis, DNA methylation, Somaclonal variation

## Abstract

Plants, known for their immobility, employ various mechanisms against stress and damage. A prominent feature is the formation of callus tissue—a cellular growth phenomenon that remains insufficiently explored, despite its distinctive cellular plasticity compared to vertebrates. Callus formation involves dedifferentiated cells, with a subset attaining pluripotency. Calluses exhibit an extraordinary capacity to reinitiate cellular division and undergo structural transformations, generating de novo shoots and roots, thereby developing into regenerated plants—a testament to the heightened developmental plasticity inherent in plants. In this way, plant regeneration through clonal propagation is a widely employed technique for vegetative reproduction. Thus, exploration of the biological components involved in regaining pluripotency contributes to the foundation upon which methods of somatic plant propagation can be advanced. This review provides an overview of the cellular pathway involved in callus and subsequent de novo shoot formation from already differentiated plant tissue, highlighting key genes critical to this process. In addition, it explores the intricate realm of epigenetic regulatory processes, emphasizing the nuanced dynamics of DNA methylation that contribute to plant regeneration. Finally, we briefly discuss somaclonal variation, examining its relation to DNA methylation, and investigating the heritability of epigenomic changes in crops.

## Introduction

Plants, as immobile land-dwelling organisms, have evolved a variety of defense mechanisms to guard against damage and stress. One understudied process is the formation of so-called callus tissue, a dedifferentiated growth of cells that develops in response to injury. Plant biology researchers have long utilized this phenomenon to grow plant tissues indefinitely in culture, representing a key model for plant physiological processes, and akin to animal cell culture.

**Fig. 1:**
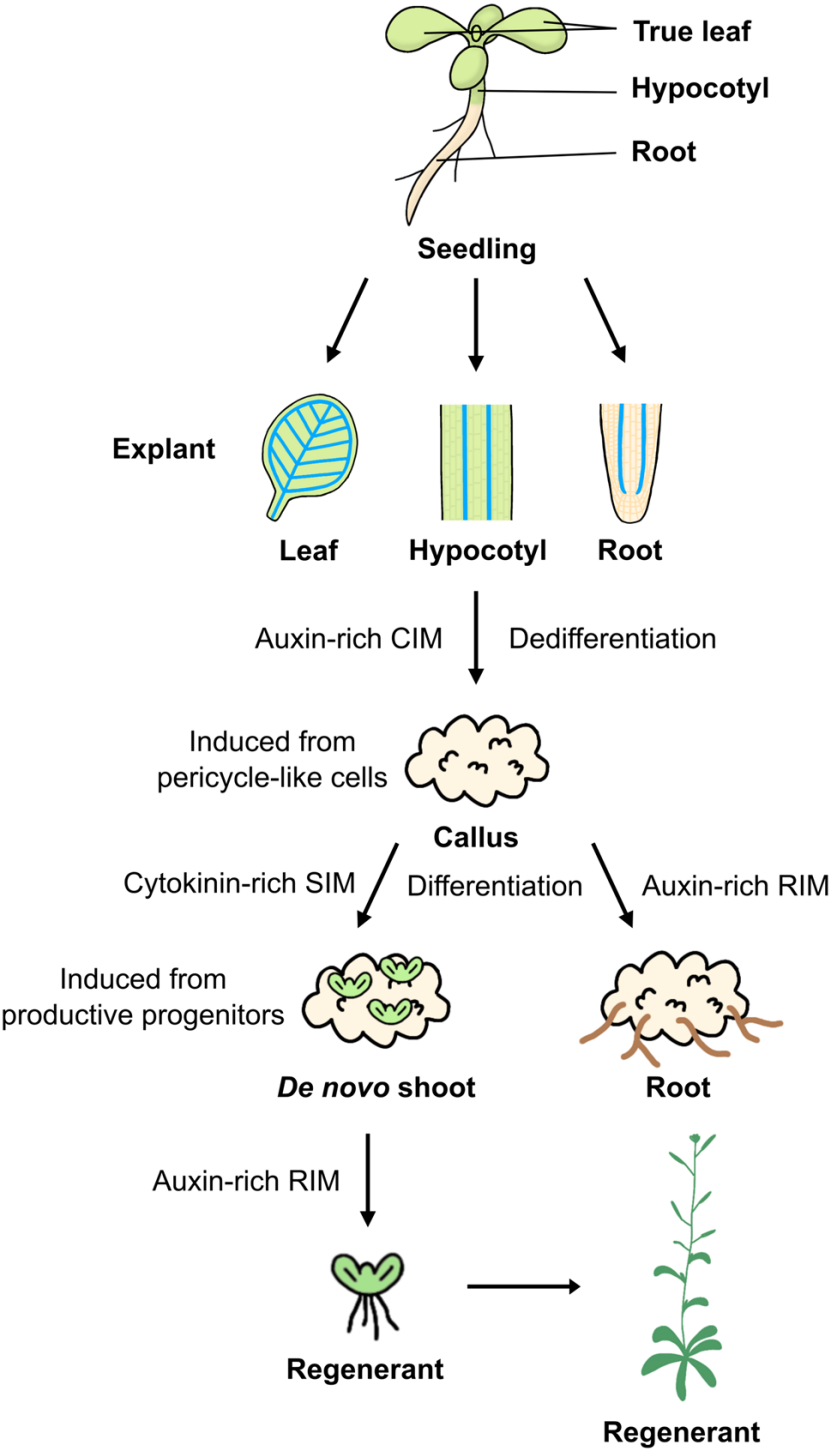
Scheme of in vitro plant regeneration. Various tissues such as true leaves, hypocotyls, and roots can be used as explants for callus induction. When the explant is incubated on an auxin-rich callus-inducing medium (CIM), dedifferentiation occurs, leading to callus induction from pericycle-like cells. Incubating the callus on a cytokinin-rich shoot-inducing medium (SIM) or an auxin-rich root-inducing medium (RIM) results in differentiation, leading to the induction of de novo shoots or roots, respectively. It has been revealed that de novo shoots are induced from the productive progenitors in the middle layer of the callus. Regenerants can be obtained by sequentially culturing the explant on CIM, SIM, and RIM

Plants exhibit a notably higher degree of natural cellular differentiation plasticity compared to vertebrates, as demonstrated by prior research (Ikeuchi et al. 2013; Sugimoto et al. 2010). When plants are subjected to stress factors such as physical injury or pathogen invasion, unorganized clusters of cells known as callus can form (Ikeuchi et al. 2013; Nagata and Takebe 1971; Steward et al. 1958). While the dedifferentiation occurring in callus tissue bears some resemblance to animal cancer, scientists have observed recovery of an almost embryo-like totipotency since the early years of callus research (Steward et al. 1958). In in vitro experiments, the induction of callus formation is achieved through various methods, including mechanical injury to plant organs or tissues and culturing plant explants on an auxin-rich callus-inducing medium (CIM). Subsequently, the induced callus can be further cultured on a cytokinin-rich shoot-inducing medium (SIM) or an auxin-rich root-inducing medium (RIM) to promote the regeneration of shoots or roots, respectively (Fig. 1) (Skoog and Miller 1957; Valvekens et al. 1988). Therefore, regenerants can be obtained by sequentially transferring newly developed shoots to a RIM to stimulate root formation.

In the past, observations have revealed that calluses possess the remarkable ability to reinitiate cellular division in previously quiescent cells and undergo structural transformations (Sugimoto et al. 2010). Consequently, it was believed that somatic cells, with a predetermined cell fate, could undergo dedifferentiation and transform into pluripotent cell masses capable of regenerating into entirely de novo shoots or roots (Kareem et al. 2016a). This is reminiscent of the in vitro reprogramming possible in mammalian cells to so-called induced pluripotency (Takahashi et al. 2007; Takahashi and Yamanaka 2006; Yu et al. 2009). In contrast to plants, however, in mammals, somatic reprogramming can only occur in the presence of exogenous signals from key transcription factors. As such, aside from the pathological cellular transformation that characterizes cancer development, reprogramming to pluripotency is not known to occur in somatic cells in vivo. These differences make plants a unique and valuable system for studying totipotency and pluripotency. In this review, we will explore the fundamental aspects of plant regeneration in the model plant *Arabidopsis* and other crop plants. In addition, we will delve into the current understanding of DNA methylation dynamics during the regeneration process. Finally, we will discuss somaclonal variation in relation to DNA methylation.

## Overview of plant regeneration: cellular diversity of callus

**Fig. 2:**
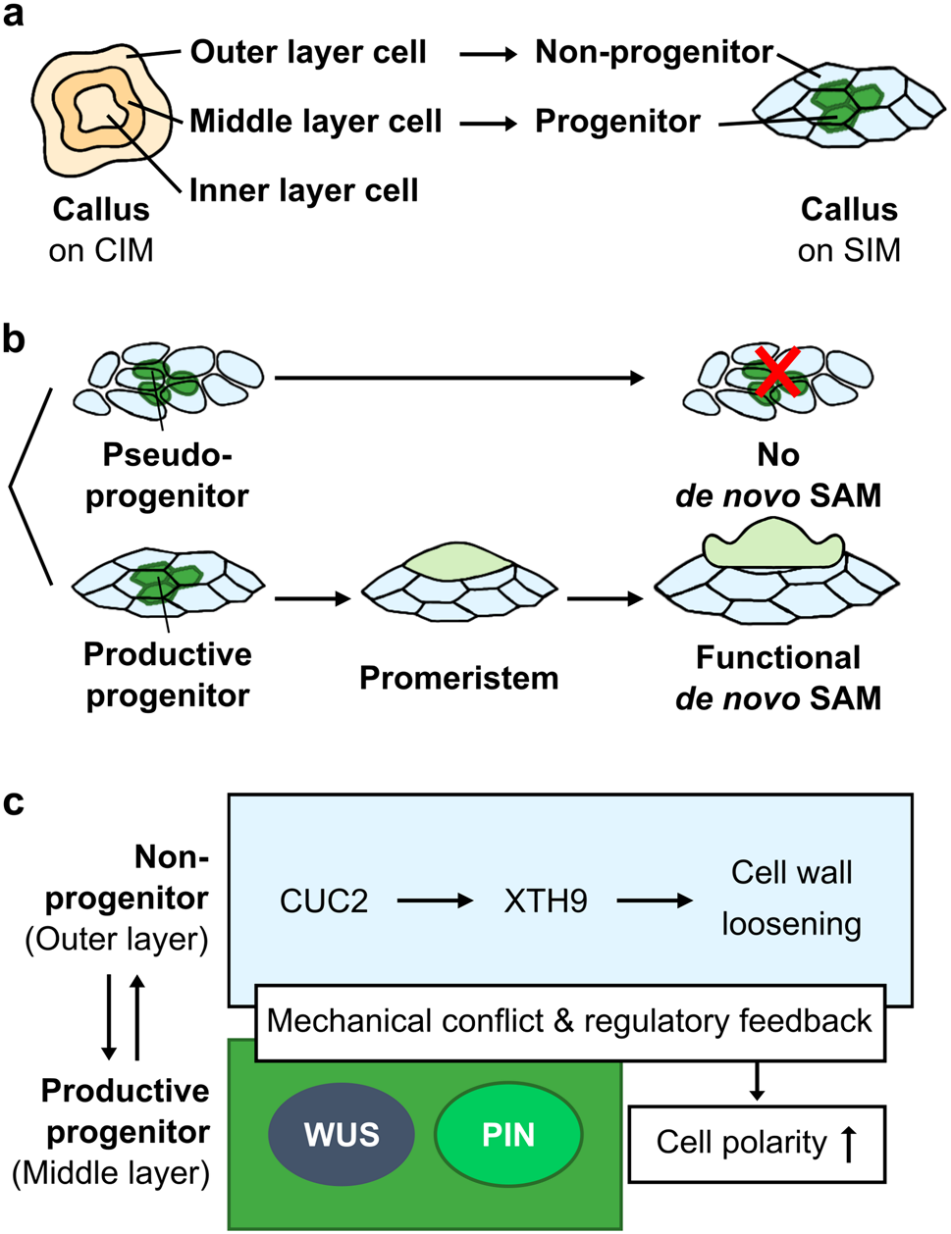
De novo shoot formation from the middle layer of callus. (**a**) The outer and middle layer cells of the callus become non-progenitor and progenitor cells, respectively, when cultured on SIM. (**b**) Progenitor cells can be further subdivided into productive progenitors which undergo a promeristem stage to generate a functional de novo shoot apical meristem (SAM), and pseudo-progenitors that fail to form a de novo SAM. (**c**) CUC2 activates XTH9 in non-progenitor cells, causing cell wall loosening. Interactions involving mechanical conflicts and regulatory feedback between the productive progenitor and adjacent non-progenitor cells contribute to the establishment of cell polarity within the productive progenitor. Consequently, the functional SAM emerges from the productive progenitor

Recent research suggests that callus formation cannot be simply characterized as a generic dedifferentiation into an undifferentiated state. Instead, it is more comparable to the formation of root meristem-like tissues regardless of explant origin (Sugimoto et al. 2010). Some researchers describe callus formation as a transdifferentiation process that enhances developmental potency (Sugimoto et al. 2011). Contrary to previous beliefs, it has been observed that callus outgrowths, initiated on CIM, do not originate from all cells of the explant but predominantly from pericycle cells located opposite the protoxylem poles (Atta et al. 2009; Sugimoto et al. 2010, 2011). The broad pluripotent potential of xylem pericycle cells was further demonstrated when their direct transfer onto media containing cytokinin resulted in the regeneration of shoot apical meristems (SAMs) from sites where lateral roots would have typically initiated (Atta et al. 2009). Furthermore, recent studies have found calluses to be a heterogeneous group of cells that are not lacking of tissue organization (Atta et al. 2009; Sugimoto et al. 2010, 2011; Zhai and Xu 2021). The clusters of callus cells were grouped into three cell layers; outer, middle, and inner layer based on their transcriptional identity (Fig. 2a) (Zhai and Xu 2021). Out of these layers, the quiescent center-like middle cell layer had *WUSCHEL RELATED HOMEOBOX 5* (*WOX5*) and *WOX7* activity which promoted TRYPTOPHAN AMINOTRANSFERASE OF ARABIDOPSIS (TAA1)-mediated auxin production. The cytokinin signaling pathway operates through a negative feedback loop involving type-A and type-B Arabidopsis Response Regulators (ARRs); type-B ARRs activate type-A ARRs, while type-A ARRs repress the signaling initiated by type-B ARRs (Buechel et al. 2010; To et al. 2004; Zhai and Xu 2021). By breaking the negative feedback loop between type-B ARR12 and type-A ARR5, WOX5 and WOX7 increase cytokinin sensitivity in the signaling pathway (Zhai and Xu 2021). This mechanism strongly suggests that the middle cell layer gains the pluripotency needed for further organ regeneration in response to hormones in CIM. Upon transfer to SIM, the shoot progenitor marker gene, *WUSCHEL (WUS)*, was induced in this layer, and cells forming adventitious shoots were shown to be descended from *WOX5*-expressing cells (Zhai and Xu 2021). Latest research has found that this middle cell layer can again be divided into sub-populations of productive progenitors that actually develop into shoot meristems and pseudo-progenitors that abort mid-way (Fig. 2b) (Varapparambath et al. 2022). The interactions between the productive progenitor and non-progenitor cells, coupled with loosening of the cell wall in non-progenitor cells by *CUP SHAPED COTELYDON 2* (*CUC2*) and *XYLOGLUCAN ENDOTRANSGLUCOSYLASE/HYDROLASE 9* (*XTH9*), induce cell polarity in productive progenitor, leading to the formation of a functional shoot apical meristem (Fig. 2c) (Varapparambath et al. 2022). This research indicates that calluses are far from being a disorganized mass and have a degree of spatial organization among the diverse cells with selective fate transition ability in certain layers.

## From genes to growth: understanding plant regenerative network

**Fig. 3:**
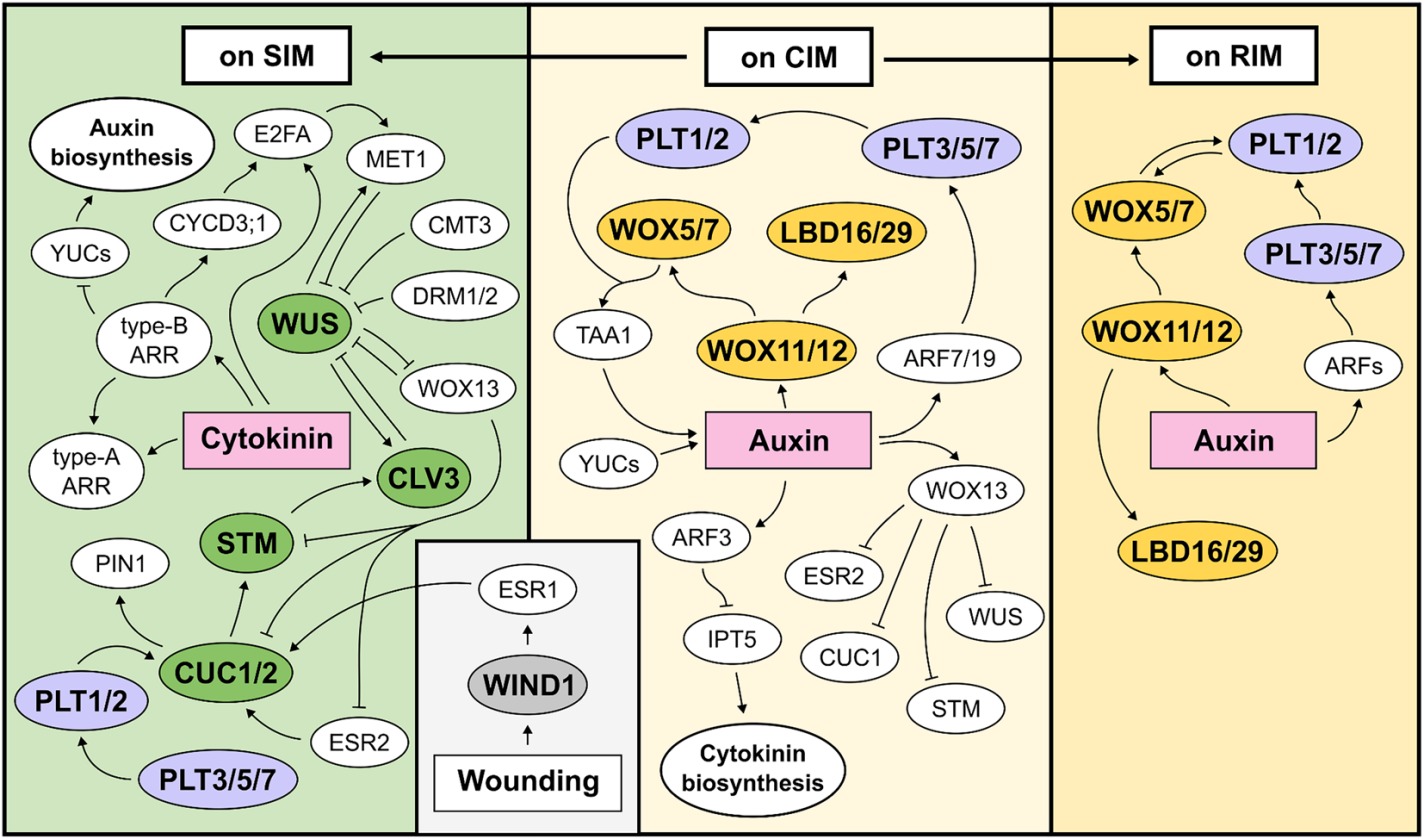
Regulatory mechanisms during plant regeneration. Wound stress induces callus formation via WIND1-ESR1 pathway and it also affects de novo shoot regeneration. The genes involved in callus formation on CIM are included in the middle box. The genes that play a role in de novo shoot formation are shown in the left box. The genes that contribute to root formation on RIM are displayed in the right box

Several critical genes have been identified to play a fundamental role in each regeneration process (Fig. 3; Table 1). Plant regeneration can be initiated by both wound stress and hormone signaling. *WOUND-INDUCED DEDIFFERENTIATION 1* (*WIND 1*) and its paralogs (*WIND2*, *WIND3*, and *WIND4*) play a crucial role in the formation of callus in response to wounding (Iwase et al. 2011, 2015). Localized wound stress triggers the expression of WIND1, which directly binds to the promoter of *ENHANCER OF SHOOT REGENERATION 1* (*ESR1*), thereby activating its expression (Iwase et al. 2017). In addition, WINDs facilitate the activation of type-B ARRs participating in cytokinin signaling, indirectly leading to the activation of *ESR1*. Thus, ESR1 promotes the formation of callus at wound sites and further influences the formation of de novo shoots through activating *CUC1* and *CUC2* (Banno et al. 2001; Iwase et al. 2011, 2017).

**Table 1:** A summary of genes involved in plant regeneration

Gene abbreviation	Full name	Function in plant regeneration	References
*ARF3*	*AUXIN RESPONSE FACTOR 3*	Transcription factor in auxin signaling; Negative regulator of de novo organ regeneration; disrupts the cytokinin biosynthesis pathway	Cheng et al. (2013)
*ARF7/19*	*AUXIN RESPONSE FACTOR 7/19*	Transcription factor in auxin signaling; Lateral root formation	Fan et al. (2012), Hofhuis et al. (2013), Okushima et al. (2007)
*CLV3*	*CLAVATA3*	Key transcriptional regulator in shoot identity acquisition	Gordon et al. (2007), Su et al. (2020)
*CMT3*	*CHROMOMETHYLASE 3*	DNA methyltransferase	Shemer et al. (2015)
*CUC1/2*	*CUP SHAPED COTELYDON 1/2*	Transcription factor in acquisition of organogenic competence	Gordon et al. (2007), Kareem et al. (2015), Ogura et al. (2023), Varapparambath et al. (2022)
*CYCD3*	*CYCLIN D3*	Cell cycle regulation	Liu et al. (2018a)
*DMC1*	*DISRUPTION OF MEIOTIC CONTROL 1*	Meiotic recombination	Doutriaux et al. (1998), Klimyuk and Jones (1997), Shim et al. (2021a)
*DRM1/2*	*DOMAINS REARRANGED METHYLTRANSFERASE 1/2*	DNA methyltransferase	Shemer et al. (2015)
*ESR1/2*	*ENHANCER OF SHOOT REGENERATION 1/2*	Transcription factor; promotes formation of callus and de novo shoots at wound sites	Banno et al. (2001), Iwase et al. (2011, 2017), Ogura et al. (2023)
*IPT5*	*ISOPENTENYLTRANSFERASE 5*	Cytokinin biosynthesis	Cheng et al. (2013)
*LBD16/29*	*LATERAL ORGAN BOUNDARIES-DOMAIN 16/29*	Transcription factor in callus formation	Fan et al. (2012), Feng et al. (2012), Hofhuis et al. (2013), Hu and Xu (2016), Liu et al. (2014, 2018b)
*MAD1*	*MITOTIC ARREST DEFICIENT 1*	Spindle assembly checkpoint	Ding et al. (2012), Shim et al. (2021a)
*MET1*	*METHYLTRANSFERASE 1*	DNA methyltransferase	Berdasco et al. (2008), Li et al. (2011), Liu et al. (2018a), Shemer et al. (2015), Shim et al. (2021b)
*ORC1*	*ORIGIN RECOGNITION COMPLEX 1*	Master subunit of origin recognition complex	Sanchez and Gutierrez (2009), Shim et al. (2021a)
*PIN1*	*PIN-FORMED 1*	Auxin efflux carrier	Gordon et al. (2007), Kareem et al. (2015)
*PLT1/2*	*PLETHORA 1/2*	Transcription factor; regulates root stem cells; enhances TAA1-mediated auxin biosynthesis with WOX5/7; establishes competence for shoot progenitor cell regeneration	Aida et al. (2004), Ding and Friml (2010), Kareem et al. (2015), Zhai and Xu (2021)
*PLT3/5/7*	*PLETHORA 3/5/7*	Transcription factor in regeneration competency acquisition; promote lateral root emergence	Hofhuis et al. (2013), Kareem et al. (2015)
*RFC2*	*REPLICATION FACTOR C 2*	Replication factor	Shim et al. (2021a)
*STM*	*SHOOT MERISTEMLESS*	Key transcription factor in shoot organogenesis initiation	Ogura et al. (2023), Su et al. (2020)
*TAA1*	*TRYPTOPHAN AMINOTRANSFERASE OF ARABIDOPSIS 1*	Auxin biosynthesis	Zhai and Xu (2021)
*type-A ARR*	*ARABIDOPSIS RESPONSE REGULATOR type-A*	Negative regulator in cytokinin signaling	Buechel et al. (2010), To et al. (2004), Zhai and Xu (2021)
*type-B ARR*	*ARABIDOPSIS RESPONSE REGULATOR type-B*	Transcription factor in cytokinin signaling	Hill et al. (2013), Meng et al. (2017), Zhai and Xu (2021)
*WIND1/2/3/4*	*WOUND-INDUCED DEDIFFERENTIATION 1/2/3/4*	Transcription factor in wound-induced callus formation	Banno et al. (2001), Iwase et al. (2011, 2015, 2017)
*WOX5/7*	*WUSCHEL RELATED HOMEOBOX 5/7*	Transcription factor; enhances TAA1-mediated auxin biosynthesis with PLT1/2; lateral root initiation	Hu and Xu (2016), Zhai and Xu (2021)
*WOX11/12*	*WUSCHEL RELATED HOMEOBOX 11/12*	Transcription factor; callus formation; reprograms initial cells into root founder cells	Hu and Xu (2016), Liu et al. (2014, 2018b)
*WOX13*	*WUSCHEL RELATED HOMEOBOX 13*	Induces cell wall modifiers; transcriptionally represses shoot meristem regulators	Ogura et al. (2023)
*WUS*	*WUSCHEL*	Key transcription factor in the initiation of shoot organogenesis	Ikeda et al. (2009), Ogura et al. (2023), Su et al. (2020), Zhai and Xu (2021)
*XTH9*	*XYLOGLUCAN ENDOTRANSGLUCOSYLASE/HYDROLASE 9*	Cell wall loosening	Hyodo et al. (2003), Varapparambath et al. (2022)
*YUC1/4*	*YUCCA 1/4*	Auxin biosynthesis	Chen et al. (2016)

Auxin and cytokinin are the two pivotal hormones in the regeneration process. High concentrations of auxin in CIM consistently and ectopically activate the expression of *WOX11* and *WOX12* (Liu et al. 2014, 2018b). This activation sequentially leads to the expression of *WOX5*, *WOX7*, *LATERAL ORGAN BOUNDARIES-DOMAIN 16* (*LBD16*), and *LBD29*, ultimately inducing callus formation during subsequent incubation on CIM through the ectopic activation of the root development pathway (Fan et al. 2012; Feng et al. 2012; Liu et al. 2014, 2018b). Auxin also triggers the activation of *AUXIN RESPONSE FACTOR 7* (*ARF7*) and *ARF19*, promoting the expression of *LBD16* and *LBD29*, thereby contributing to callus induction. In addition, these ARFs regulate *PLETHORA3* (*PLT3*), *PLT5*, and *PLT7*, which function downstream in auxin-mediated lateral root initiation within pericycle cells (Hofhuis et al. 2013). PLT3/5/7 leads to the induction of maintenance regulators of root stem cells, specifically PLT1 and PLT2 (Kareem et al. 2015). Another auxin response factor ARF3 functions as a negative regulator of de novo organ regeneration by directly binding to the *ISOPENTENYLTRANSFERASE 5* (*IPT5*) promoter, disrupting the cytokinin biosynthesis pathway under high auxin concentration conditions (Cheng et al. 2013). Furthermore, epigenetic modifications such as H3K27me3 are also apparent when it comes to transcriptional repression of regeneration-associated genes (He et al. 2012; Ikeuchi et al. 2015; Lafos et al. 2011; Mozgova et al. 2015; Yan et al. 2020). Particularly, the complex POLYCOMB REPRESSIVE COMPLEX 2 (PRC2), a histone methyltransferase known to be crucial in developmental transition, increases H3K27me3 marks at loci of genes such as WINDs, PLT1, PLT2, WOXs, YUCCAs (YUCs), and WUS (Bemer and Grossniklaus 2012; Ikeuchi et al. 2015; Xiao et al. 2017). On CIM, WOX13, a negative regulator of shoot regeneration, is upregulated by auxin. WOX13 induces cell wall modifiers as well as transcriptionally represses shoot meristem regulators such as WUS, SHOOTMERISTEMLESS (STM), ESR2, and CUC1 (Ogura et al. 2023).

De novo organogenesis from callus can be categorized into shoot regeneration and root regeneration (Fig. 1), with the determining factors being the balance of auxin and cytokinin, along with their associated genes (Che et al. 2006; Feldmann and Marks 1986; Liu et al. 2018b; Skoog and Miller 1957; Zhao et al. 2013). Callus, the lateral root meristem (LRM)-like structure, can subsequently be reprogrammed into SAMs when transferred onto SIM. The presence of LRM or LRM-like primordia is considered a prerequisite for de novo shoot formation (Sugimoto et al. 2010). *PLT3, PLT5*, and *PLT7* establish competence for regenerating shoot progenitor cells by inducing root stem cell regulators *PLT1* and *PLT2*. Consequently, CUC2 is upregulated. It is noteworthy that as described earlier, CUC2 is also activated by the WIND1-ESR1 pathway. Thus, CUC2 serves as a key regulator in the initiation of shoot formation, bridging the WIND1 and PLT pathways and PIN-FORMED 1 (PIN1) induced by CUCs determines the future location of shoot progenitors (Gordon et al. 2007). In the early stages on SIM, METHYLTRANSFERASE 1 (MET1), induced by the cytokinin-CYCLIN D3 (CYCD3)-E2FA module, represses WUS expression, thereby preventing the transition of cells into shoot cells and maintaining the identity of callus (Liu et al. 2018a). Other DNA methylation-related genes *CHROMOMETHYLASE 3* (*CMT3*) and *DOMAINS REARRANGED METHYLASE 1* (*DRM1*) and *DRM2* also repress the expression of WUS (Shemer et al. 2015). During further incubation on SIM, the region expressing MET1 turns into the outer cell layers of the callus, while WUS is activated by the type-B ARRs beneath the MET1-expressing regions (Liu et al. 2018a). WUS interacts with STM, a transcription factor crucial for the proliferative state of meristematic cells, enhancing its binding to the *CLAVATA3* (*CLV3*) promoter and promoting CLV3 expression. Both WUS and STM are key transcription factors in the initiation of shoot organogenesis (Su et al. 2020). On SIM, WUS represses WOX13, enabling the operation of shoot meristem regulators including STM, ESR2, and CUC1. Simultaneously, this repression lessens the inhibition of WUS by WOX13 (Ogura et al. 2023). Recent research has also indicated the potential for direct regeneration, wherein shoot apical meristems can develop directly from root explants, bypassing the callus stage (Kareem et al. 2016b; Rosspopoff et al. 2017).

In the process of de novo root regeneration on RIM, auxin initiates the expression of *WOX11* and *WOX12*, activating a cascade involving *WOX5, WOX7*, and *LBD16*, which collectively contribute to the division of root founder cells and their subsequent transition into root primordium cells (Liu et al. 2014, 2018b). LBD16 is expressed in dividing root founder cells and developing root primordia but diminishes during the establishment of the root meristem, while WOX5 is confined to the stem cell niche in the emerging root apical meristem (RAM) (Hu and Xu 2016). The genes *PLT1* and *PLT2*, whose transcription relies on auxin accumulation and ARFs, play a crucial role in specifying the quiescent center and maintaining stem cell activity in the RAM (Aida et al. 2004). WOX5 is essential for PLT1 expression in RAMs (Ding and Friml 2010).

Beyond the pivotal changes in key gene expression, recent reports are progressively unveiling the critical role of epigenetic reprogramming, encompassing histone modifications and DNA methylation, in the induction of regenerative competency throughout the regeneration process. Our primary focus will be exploring the DNA methylation dynamics during regeneration in *Arabidopsis* and other crop plants.

## DNA methylation dynamics during *Arabidopsis* regeneration

**Fig. 4:**
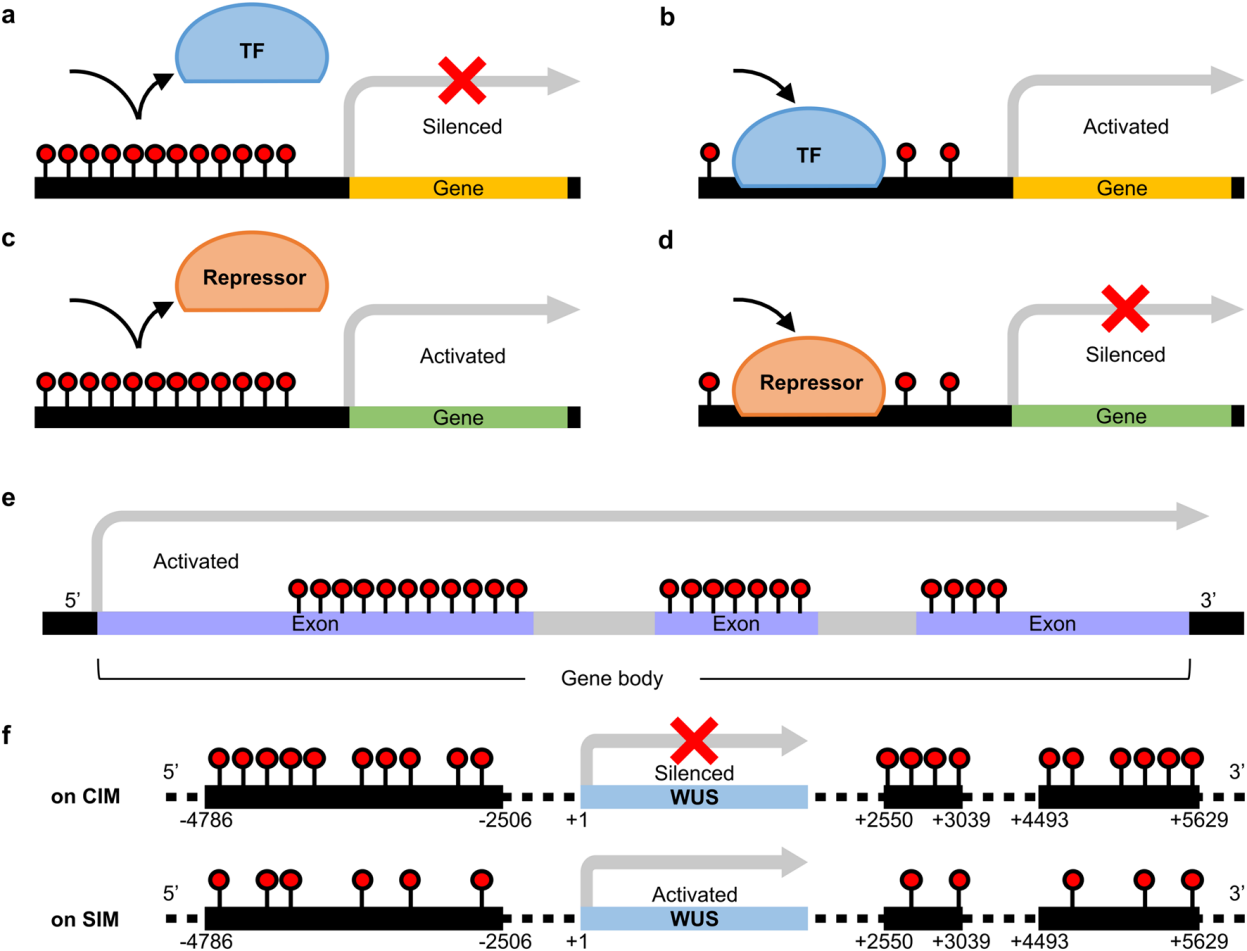
Regulation of gene expression via DNA methylation. (**a**–**d**) Promoter methylation. While promoter methylation is widely recognized for its role in silencing gene expression (**a**, **b**), in some instances, methylation in the promoter region can promote gene expression. This possibly occurs as methylation acts to inhibit the binding of repressor proteins (**c**, **d**). (**e**) Gene body methylation. Gene body methylation (GbM) is most commonly observed in constitutively expressed genes, often referred to as housekeeping genes. Although the function of GbM remains largely unknown and is still a subject of debate, one plausible role is maintaining homeostasis (Zilberman 2017). This may involve preventing aberrant transcription from internal cryptic promoters within the gene body or enhancing splicing efficiency. (**f**) Methylation at the 5′-upstream and 3′-downstream regions of the *WUS* gene influences gene expression

DNA methylation, characterized by the addition of a methyl group to the 5th carbon of cytosine, stands as a crucial epigenetic modification with pivotal roles in transposon silencing and gene regulation. The latter often depends on the specific position of the methylated region in regard to the gene (Fig. 4). DNA methylation processes can be categorized into three distinct phases: establishment, maintenance, and removal (Law and Jacobsen 2010). These stages involve specific enzymes and pathways that ensure the accurate inheritance of epigenetic marks during cell division and differentiation, and exhibit conservation with the process in animals. However, whereas DNA methylation primarily occurs in the CG context in animals, three cytosine contexts can be methylated in plants: CG, CHG, and CHH (where H = A, T, or C). The de novo establishment of methylation in plants occurs through the RNA-directed DNA methylation (RdDM) pathway, regardless of context. DRM2 plays a crucial role in this process. For maintenance of methylation after establishment, MET1 is involved in the CG context, CMT3 is involved in the CHG context, and CMT2, DRM1, and DRM2 are involved in the CHH context (Grimanelli and Ingouff 2020; Law and Jacobsen 2010; Yaari et al. 2019). DNA demethylation can be divided into passive and active demethylation. Passive demethylation arises due to imperfect maintenance of methylation during replication, while active demethylation is driven by the action of DNA demethylases. Active demethylation takes place through the DNA base-excision repair pathway, facilitated by DNA glycosylase-domain protein, including REPRESSOR OF SILENCING 1 (ROS1), DEMETER (DME), DME-LIKE 2 (DML2), and DML3 in *Arabidopsis* (Choi et al. 2002; Gong et al. 2002; Liu and Lang 2020; Ortega-Galisteo et al. 2008; Penterman et al. 2007).

*Arabidopsis*, as a model plant, has offered invaluable insights into the role of DNA methylation in plant regeneration. During the transition from leaf to callus, it has been observed that average CG methylation remains relatively stable, while CHG methylation increases and CHH methylation decreases, with these changes being particularly enriched in transposable elements (TEs) (Shim et al. 2021a). Interestingly, these changes in CHG and CHH methylation levels correspond with alterations in the expression of their respective enzymes. Notably, during the callus formation process, an upregulation of *CMT3* and a downregulation of *CMT2* were observed (Lee et al. 2016).

MET1-mediated DNA methylation in callus exerts a negative regulatory influence on the activity of key genes involved in shoot regeneration, such as *WUS*, thereby restricting the initiation of de novo shoot organ development (Li et al. 2011; Liu et al. 2018a). However, when shoots are initiated from callus, there is a reduction in global DNA methylation levels. This decrease in DNA methylation levels induces *WUS* transcription, ultimately accelerating shoot formation (Berdasco et al. 2008; Li et al. 2011; Shemer et al. 2015). Consistent with this, more shoots were generated from *met1* mutant callus when transferred to SIM (Shim et al. 2021b).

The regeneration capacity of *Arabidopsis* can be influenced not only by global changes in methylation levels, but also by alterations in the methylation levels of individual genes associated with regeneration. Interestingly, several key genes associated with pluripotency, cell proliferation and replication, such as *PLT1, PLT2, ORIGIN RECOGNITION COMPLEX 1* (*ORC1*), *REPLICATION FACTOR C 2* (*RFC2*), *MITOTIC ARREST DEFICIENT 1* (*MAD1*), and *DISRUPTION OF MEIOTIC CONTROL 1* (*DMC1*), exhibited transcriptional upregulation, along with CHH hypomethylation, during callus induction (Shim et al. 2021a). Therefore, it is tempting to speculate that dynamic methylation changes during callus formation might activate genes for pluripotency acquisition and cell proliferation. Since *Arabidopsis* regeneration involves intricate DNA methylation dynamics with context-specific patterns influencing gene expression and cell differentiation, investigating these epigenetic changes will shed light on the molecular mechanisms underlying plant regeneration.

## Impact of DNA methylation pathway gene mutations on *Arabidopsis* regeneration

It has been consistently reported that alterations in DNA methylation can lead to varying degrees of regeneration, suggesting that changes in DNA methylation alone can potentially influence the callus induction and de novo shoot formation (Table 2). In the *met1* mutant, there was a lower induction of callus compared to the wild type (Berdasco et al. 2008). Promoter hypermethylation and transcriptional repression in some key genes of undifferentiated calluses were altered in *met1* mutants (Berdasco et al. 2008). This is consistent with another study showing a slight decrease followed by an increase in *MET1* expression during wild type callus formation (Shim et al. 2021a). It is plausible that *met1*-mediated hypomethylation could lead to the transcriptional de-repression of negative regulator for callus formation or, could create a new target site for PRC2 or repressor binding, potentially leading to the silencing of the activator (Fig. 4).

**Table 2:** Regeneration phenotypes of methylation-related mutants of *Arabidopsis*

Affected protein name (Protein ID)	Mutant name	Original function	Regeneration phenotype	Ecotype	References
MET1 (AT5G49160)	*met1*	DNA methyltransferase (CG)	Reduced callus induction (size)	Ws	Berdasco et al. (2008)
MET1 (AT5G49160)	*met1*	DNA methyltransferase (CG)	Precocious shoot induction; frequency^a^ of shoot induction unchanged at 18 days	Ws	Li et al. (2011)
MET1 (AT5G49160)	*met1-3*	DNA methyltransferase (CG)	Increased shoot induction at 3 weeks (number per callus)	Col	Shim et al. (2021b)
MET1 (AT5G49160)	*MET1*-overexpressing (*MET1*-OE)	DNA methyltransferase (CG)	Decreased shoot induction (frequency^a^, number per callus)	Col	Liu et al. (2018a)
CMT3 (AT1G69770)	*cmt3*	DNA methyltransferase (non-CG)	Enhanced callus induction (size)	Ws	Berdasco et al. (2008)
CMT3 (AT1G69770)	*cmt3-11*, *cmt3-7*	DNA methyltransferase (non-CG)	High ability of shoot induction on SIM-direct	Col-0 (*cmt3-11*), Ws (*cmt3-7*)	Shemer et al. (2015)
DRM2 (AT5G14620)	*drm2*	DNA methyltransferase	Reduced callus induction (size)	Ws	Berdasco et al. (2008)
DRM1, DRM2 (AT5G15380, AT5G14620)	*drm1/drm2* double mutant	DNA methyltransferase, DNA methyltransferase	Enhanced callus induction (size, weight)	Ws	Jiang et al. (2015)
DRM1, DRM2, CMT3 (AT5G15380, AT5G14620, AT1G69770)	*drm1/drm2/cmt3-11* triple mutant	DNA methyltransferase, DNA methyltransferase, DNA methyltransferase (non-CG)	High ability of shoot induction on SIM-direct	Col-0	Shemer et al. (2015)
DME (AT5G04560)	*dme-2*	DNA glycosylase	Enhanced callus induction (weight); increased shoot induction (number per callus)	Ler	Kim et al. (2021)

^a^Frequency was calculated as the number of shoot-producing calluses divided by the total number of calluses cultured on SIM

During the de novo shoot formation process, *WUS*, the organizing center regulator, is induced earlier in the *met1* shoots compared to the wild type. As a result, shoot induction occurs more precociously in *met1* than in the wild type (Li et al. 2011). Even though more shoots were induced in *met1* mutant calluses (Shim et al. 2021b), the ratio of shoot-producing calluses to the total number of calluses cultured on SIM was similar to that of the wild type (Li et al. 2011). Similarly, the number of induced shoots from *met1* mutants remained relatively unchanged in prolonged SIM conditions at 18 days (Li et al. 2011). Intriguingly, the shoot regeneration phenotype of a *MET1*-overexpressing line showed a decrease in both the number of shoots per calluses and the ratio of shoot-producing calluses (Liu et al. 2018a). Adding another layer of complexity, cell cycle regulators E2FA and CYCD3 activate *MET1* expression. Despite MET1’s known role in repressing *WUS*, mutants of *e2fa* or *cycd3* result in reduced *WUS* expression (Liu et al. 2018a). This implies that *WUS* expression might be regulated not only by MET1, but also by other cell cycle targets. Further studies are needed to elucidate the precise molecular mechanisms underlying genetic network associated with MET1-mediated DNA methylation effects during the regeneration process*.*

CMT3 is required for maintaining CHG methylation. Despite an increase in CHG methylation levels during wild type leaf to callus transition, more calluses were induced in *cmt3* mutants (Berdasco et al. 2008). Interestingly, root explants from *cmt3–11* and *cmt3–7* mutants that were placed directly on SIM without preincubation on CIM exhibited a high ability to regenerate shoots compared to wild type (Shemer et al. 2015). By contrast, the expression of *DRM2*, a de novo methyltransferase, gradually decreases during the initial stages of callus induction (Shim et al. 2021a). Callus induction was reduced in *drm2* mutants but enhanced in *drm1/drm2* double mutants, possibly due to their redundancy (Berdasco et al. 2008; Jiang et al. 2015). Direct organogenesis, as observed in *cmt3* mutants, was also noted in *drm1/drm2/cmt3* (*ddc*) triple mutants. While wild type explants directly incubated on SIM showed no endogenous *WUS* expression, those subsequently incubated on CIM-SIM, as well as *ddc* explants directly incubated on SIM, exhibited *WUS* expression, which is essential for shoot regeneration. This suggests that the reduction of non-CG methylation in the *ddc* mutant enabled *WUS* expression in response to SIM and implies that DNA methylation plays a critical role in the regulation of *WUS* expression (Shemer et al. 2015). In summary, it is plausible that the loss of non-CG DNA methylation can enhance callus induction and direct shoot organogenesis.

DME, a DNA glycosylase that removes DNA methylation as part of the base-excision repair (BER) pathway, also influences callus and de novo shoot formation (Kim et al. 2021). During callus induction, the expression of *DME* diminishes over time (Shim et al. 2021a). Accordingly, more calluses were generated in *dme* mutants compared to wild type (Kim et al. 2021), which is opposite to the *met1* mutant phenotype which shows less callus formation. Furthermore, de novo shoot formation of *dme* mutants was significantly increased compared to wild type L*er* (Kim et al. 2021). When comparing the *dme-2* transcriptome to wild type, numerous genes related to regeneration, such as *LBD16, PLT1, PLT2, PLT5, WOX4, WOX5, WOX12*, and *WOX14*, exhibited significantly higher expression levels (Kim et al. 2021). It is tempting to speculate that DME directly activates negative regulators of cell division or regeneration via DNA demethylation, so that more cell proliferation can be observed in *dme* calluses or de novo shoots. Interestingly, the cellular overproliferation phenotype was first reported in the *dme* mutant endosperm (Choi et al. 2002). The phenotype observed in *dme* mutants during regeneration cannot be solely attributed to DNA methylation; it may involve more complex control mechanisms, such as RNA-directed DNA methylation and downstream polycomb activities. In-depth analyses, incorporating transcriptome, methylome, and small RNAome studies using these methylation mutants in conjunction with *dme* mutants, will provide insights into the effects of DNA methylation and demethylation on the regeneration process.

## Generation of somaclonal variation during regeneration

The term somaclonal variation, first coined by Larkin and Scowcroft, is used to describe variants within tissue-cultured plants that arise from the varying weights of contributions from sequence-level modifications and epigenetic changes (Bairu et al. 2011; Larkin and Scowcroft 1981). Regenerants often show strong variation which can be hard to pinpoint to a few singular causal factors. Studies have found factors ranging from explant origin to conditional factors such as hormone concentration, light condition, and temperature to influence regeneration (Ikeuchi et al. 2016; Nameth et al. 2013; Sugimoto et al. 2010). A shoot regeneration study of multiple *Arabidopsis* genotypes found that the strong variation within the same genotype was likely due to environment, physiological state of explants, and epigenetic effects (Lardon et al. 2020). Other than shoot regeneration, multiple morphological differences ranging from root-like outgrowths to structures resembling leaves or flower buds were also observed (Lardon et al. 2020). When it comes to the genes that potentially cause these variations, multiple loci including novel candidate genes such as *EMBRYO SAC DEVELOPMENT ARREST 40* (*EDA40*), *DNA-BINDING WITH ONE FINGER 4.4* (*DOF4.4*) and *AT3G09925* were identified at various de novo shoot organogenesis stages for the regulation of regeneration traits in a context-dependent manner (Lardon et al. 2020).

Differences in DNA methylation, in conjunction with genetic variations, have been identified as a significant and recurring source of somaclonal variation in regenerants (Coronel et al. 2018; Kaeppler et al. 2000). While opinions may vary on whether DNA methylation is the primary causal factor of variation, regenerated plants do indeed exhibit significantly different methylation patterns. These variations include a greater diversity and a higher number of transposable elements with non-fully methylated flanking regions, which could potentially enhance their mobilization (Coronel et al. 2018; Jiang et al. 2011).

## DNA methylation changes during crop regeneration

In addition to studies on *Arabidopsis*, crop plants such as maize and rice are of significant importance due to their direct relevance for commercial use. In maize, an increase in DNA methylation associated with small RNA expression was observed during embryo tissue culture, resulting in the formation of embryo-derived calluses (Liu et al. 2017). These changes were found to be absent in the pre-callus induction maize embryo cell population, suggesting that the DNA methylation alterations specific to callus formation were not merely a consequence of the expansion of a few cell types into embryonic calluses. While the focus is primarily on embryo dedifferentiation rather than re-differentiation, it is worth noting that genetic manipulation through the former process frequently precedes callus formation during plant regeneration. The results of such manipulation were consistently considered to be indicative of epigenetic changes during tissue culture, which could manifest as phenotypic variations in regenerated maize (Liu et al. 2017).

Tissue-cultured regenerated plants also showed epigenomic changes, with rice and triticale showing an overall tendency of DNA methylation loss, and barley showing the opposite (Machczynska et al. 2014; Orlowska et al. 2016; Stroud et al. 2013). In rice, CG hypomethylation differentially methylated regions (DMRs) were enriched in regenerated plants and different sites of the genome displayed differential susceptibility to loss of methylation (Stroud et al. 2013). Loss of non-CG methylation was also observed with it generally being associated with CHG rather than CHH methylation. In the case of CHH hypomethylation DMRs, loss of 24-nt siRNAs was suggested as the likely reason behind their absence as the typical enrichment of 24-nt siRNAs at CHH methylated areas was absent in regenerated plants (Stroud et al. 2013). This suggestion is reinforced in grapevine embryogenic callus, in which the accumulation of CHH methylation was correlated with an abundance of TE transcripts and corresponding 24-nt siRNAs (Lizamore et al. 2021).

DNA methylation changes are particularly enriched in the promoter region and lead to altered expression of certain genes in both maize and rice (Liu et al. 2017; Stelpflug et al. 2014; Stroud et al. 2013). In rice, these genes were not closely connected to specific biological processes, suggesting a seemingly random pattern (Stroud et al. 2013). Deregulation of these genes mostly resulted in higher expression. In particular, Stroud et al. observed that the closer the hypomethylation DMR was to the gene transcription start site, the more deregulated the genes were (Stroud et al. 2013). In addition to changes in gene expression, retrotransposon activity was also altered in tissue-cultured rice (Zhang et al. 2014). Cytosine methylation remodeling during and after tissue culture was detected in the 5′-Long Terminal Repeats (LTRs) of Tos17, the most active retrotransposon in rice (Zhang et al. 2014). This indicates that TE repression may have been compromised as hypermethylation at the 5′LTRs of retrotransposons is thought to reflect silencing through the RdDM pathway (Zhang et al. 2014; Cheng et al. 2006). However, the mechanism behind the alteration of TE activity by tissue culture cannot solely be attributed to DNA methylation, as there were cases in which the correlation between DNA methylation changes and TE activity was strong in one and weak in another (Cheng et al. 2006). This viewpoint is further reinforced in barley as an increase in global DNA methylation did not correspond to TE stabilization (Orlowska et al. 2016). Deregulation in genes and TE highlight the potential alteration of repressive epigenetic traits in plant tissue culture. Future examination of their phenotypic implications is crucial to understand the extent of the impact that tissue culture process may have.

## Heritability of epigenomic changes and somaclonal variation in regenerated plants

A methylome study of calluses, regenerants, and regenerant-derived progeny in maize demonstrated the heritability of DNA methylation changes arising from tissue culture processes (Stelpflug et al. 2014). Han et al. also observed heritable epigenomic changes in maize (Han et al. 2018). While only around 30% of CG and CHG context methylation changes in calluses were transmitted to primary regenerants, the majority of the CG and CHG methylation changes observed in primary regenerants were inherited by progeny. This suggests that only a subset of methylation changes induced during tissue culture are heritable to regenerants. It is also worth noting that there were context-wise differences in consistent DMRs, which are those shared by more than 50% of samples. While a significant portion of CG and CHG DMRs exhibited consistency among regenerant samples, 99% of CHH methylation changes were not consistent (Han et al. 2018). Context-wise peculiarities in methylation patterns were also observed in various plant backgrounds (Stroud et al. 2013; Liu et al. 2022; Wang et al. 2022). In rice, while most CG hypomethylations in regenerated plants were stable through generations, hypermethylation that occurred at the callus stage was completely lost in regenerated plants (Stroud et al. 2013). This hypermethylation was specifically in the CHH context, mostly corresponding to promoter regions and showing high coincidence between two callus samples (Stroud et al. 2013). These outlying tendencies of CHH methylation are intriguing since CHH methylation is asymmetric and patterns can only be recapitulated through signaling and the presence of guiding histone modifications. In triticale, an onset of reestablishment of decreased DNA methylation was observed in the third generation of progeny (Machczynska et al. 2014). However, whether this leads to a full recovery of tissue culture-induced demethylation in future generations remains unexplored.

In addition to the heritability of differential methylation, notable variation of differential methylation was observed between cell culture lines of maize, with no differential methylation detected between the non-cultured sibling control plants (Stelpflug et al. 2014). Overall, hypomethylation events were more prevalent than hypermethylation events, and hypomethylated regions were consistently found in independent regenerants. When examining the DMRs in calluses derived from maize embryos across four independent cell culture lines of the same maize plant, hypermethylated DMRs were predominantly observed in only one cell line (Stelpflug et al. 2014). This observation suggests that hypermethylated DMRs were more likely to occur randomly or stochastically when compared to hypomethylated DMRs. Interestingly, the degree of overlap between culture-derived DMRs and naturally occurring DMR profiles was greater than would be expected by chance alone (Stelpflug et al. 2014). Han et al. also found that certain loci are more prone to epigenetic variation (Han et al. 2018). In rice, Stroud et al. found that certain sites were more susceptible to methylation change and recurred among regenerated lines (Stroud et al. 2013). This suggests the presence of specific genomic loci that are particularly susceptible to epigenetic changes when exposed to stress factors. These findings increasingly support the notion that phenotypic changes coming from epigenetic variations are a valuable source to draw from when cultivating crops in relation to various environmental stress factors.

## Enhancing efficiency in plant regeneration and propagation

Many factors involved in plant regeneration efficiency have been identified. First, preparing media with the appropriate ratio of auxin to cytokinin is significantly important for enhancing regeneration efficiency. Using a combination of different types of auxins, such as 2,4-dichlorophenoxyacetic acid (2,4-D) and 1-naphthaleneacetic acid (NAA), can also enhance callus formation (Din et al. 2016). In addition, regulating the expression of type-B ARR to enhance cytokinin sensitivity can be utilized. ARR10 is particularly stable among type-B ARR and it is confirmed that expression of ARR10 driven from the ARR1 promoter enhances callus formation and shoot regeneration in *Arabidopsis* (Hill et al. 2013). Trichostatin A (TSA) is a histone deacetylase inhibitor, and the acetylation induced by TSA enhances the regeneration potential in certain barley genotypes (Nowak et al. 2024). Regulating the concentration of amino acids, such as tryptophan and glutamine, can also influence the induction performance (Din et al. 2016). Copper also plays a crucial role in enhancing regeneration efficiency. An optimal concentration of copper significantly improved the induction of callus and formation of shoots and roots in various plants, including barley, sorghum, wheat, and triticale (Dahleen 1995; Nirwan and Kothari 2003; Purnhauser and Gyulai 1993). Further research to enhance plant regeneration efficiency will directly contribute to increased crop yields.

Understanding the functional relevancy of DNA methylation in crop phenotypes and yield can provide insight into how epigenomic alterations can enhance propagation. In large-seeded chickpea, several seed size- and -weight related genes showed CG context hypermethylation within the gene (Rajkumar et al. 2020). Methylation in the CG context plays a crucial role in the seed development of *Arabidopsi*s. Global demethylation, mediated by the *met1* mutation in the CG context but not in the CHG or CHH contexts, leads to distinct seed size difference depending on parental origin when crossed with wild type (Xiao et al. 2006; Lin et al. 2017). These observations suggest DNA methylation in the CG context to be a point of focus when it comes to altering seed phenotypes for propagation. Plants dynamically modulate DNA methylation in response to environmental and developmental stresses, introducing another avenue of exploration when leveraging epigenomic modifications to achieve desirable crop traits. Investigating epigenomic changes during these stresses will unveil potential sources of crop trait variation, while also providing insights into specific regulatory pathways that can be effectively modulated in response to stress factors.

## Conclusions and perspectives

Plant regeneration through clonal propagation is a frequently utilized method of vegetative propagation, representing a form of asexual reproduction. In theory, this method allows for the inheritance of beneficial genetic or epigenetic traits that are already present, bypassing the need for sexual reproduction. As this, in turn, can lead to improved crop yields, harnessing propagation techniques is a cornerstone of modern horticulture. Thus, understanding the intricacies of callus formation and the subsequent de novo shoot and root formation leading to regenerated plants is crucial. Despite being asexually reproduced from the same explant origin, calluses, shoots, and regenerants have exhibited unexpected vibrancy with varying gene expression and epigenetic states. Consequently, it is essential to gain a deeper understanding of the epigenetic processes governing regeneration. This is especially so, as the resulting variation can both be a source of divergence to draw from, and also a factor that needs to be controlled for consistent propagation of desirable traits. However, the molecular and hereditary mechanisms underlying these processes remain incompletely understood.

The study of plant regeneration has long served as a versatile and essential model for investigating plant physiology in highly controlled in vitro environments. Yet, the study of callus itself holds immense value in the realm of plant regeneration and adaptation. It contributes not only to our understanding of the fundamental biology behind regaining pluripotency but also sheds light on the crucial advantages conferred upon sessile plants for their survival. By uncovering the epigenetic processes that underlie the reprogramming of plant calluses, both in vitro and in vivo, we can begin to unravel the genetic and epigenetic pathways crucial to this vital process, paving the way for the development of optimized somatic plant propagation strategies.

## Data Availability

No datasets were generated or used for this review paper.
